# The application of nasoseptal “rescue” flap technique in endoscopic transsphenoidal pituitary adenoma resection

**DOI:** 10.1007/s10143-018-1048-8

**Published:** 2018-12-11

**Authors:** Chao Zhang, Ning Yang, Long Mu, Chunxiao Wu, Chao Li, Weiguo Li, Shujun Xu, Xingang Li, Xiangyu Ma

**Affiliations:** 1 Department of Neurosurgery, Qilu Hospital, Shandong University, 107 Wenhua Western Rd., Jinan, 250012 Shandong China; 2 Brain Science Research Institute, Shandong University, 44 Wenhuaxi Road, Jinan, China; 3 Department of Anesthesiology, Zhangqiu People Hospital, Jinan, China

**Keywords:** Pituitary adenoma, Endoscopic, Transsphenoidal, Nasoseptal “rescue” flap, Nasal septum flap

## Abstract

To explore the reliability and superiority of nasoseptal “rescue” flap technique in neuroendoscopic transnasal pituitary adenoma resection. Retrospective clinical analysis of 113 cases of endoscopic transsphenoid pituitary adenoma resection with the application of nasoseptal “rescue” flap technology. The reliability and the superiority of the technique were evaluated according to the duration of nasal cavity and sphenoid sinus stage, the incidence of postoperative anosmia, and cerebrospinal rhinorrhea. The duration of nasal and sphenoid sinus stage was 15–30 min, averaging 24 min. There were 27 cases of intro-operative cerebrospinal fluid leakage, including 24 cases of low-flow cerebrospinal fluid leak and 3 cases of high-flow cerebrospinal fluid leak. Twenty-three cases were converted from nasoseptal “rescue” flap to nasal septum flap. There were 17 cases of postoperative olfactory decline or disappearance, 1 case of epistaxis and 1 case of cerebrospinal rhinorrhea. The application of nasoseptal “rescue” flap technique can proceed sellar floor reconstruction when the diaphragma sellae rupture occurs during the operation. There is no obvious increase of the duration of sphenoid sinus and nasal stage and the rate of postoperative olfactory loss. This technique can be used as a conventional technique for endoscopic transsphenoid pituitary adenoma resection.

## Background

Cerebrospinal fluid rhinorrhea is one of the most common complications of surgical treatment of pituitary adenoma under neuroendoscope. However, the incidence of this complication was greatly reduced by using the technique of pedicled nasoseptal flap (usually originated from mucoperiosteum and mucoperichondrium of the nasal septum), which established the technical foundation for the reconstruction of the skull base for the neuroendoscopic surgery [1, 2]. At present, the commonly used methods in the treatment of sphenoid sinus mucosa cannot effectively transfer to the nasal septum mucosa under the condition of cerebrospinal fluid rhinorrhea. Rivera-Serrano [3] reported the application of rescue flap technique to treat the septum mucosa of the nasal septum of the sphenoid sinus; the submucosal flap of the nasal septum was reconstructed in the case of cerebrospinal fluid leakage in operation. The neurosurgery department of Shandong University of Qilu Hospital applied this technique to perform 113 cases of neuroendoscopic transsphenoidal approach adenoma resection, which presented an optimistic result. The present report is as follows:

## Methods and materials

**Fig. 1:**
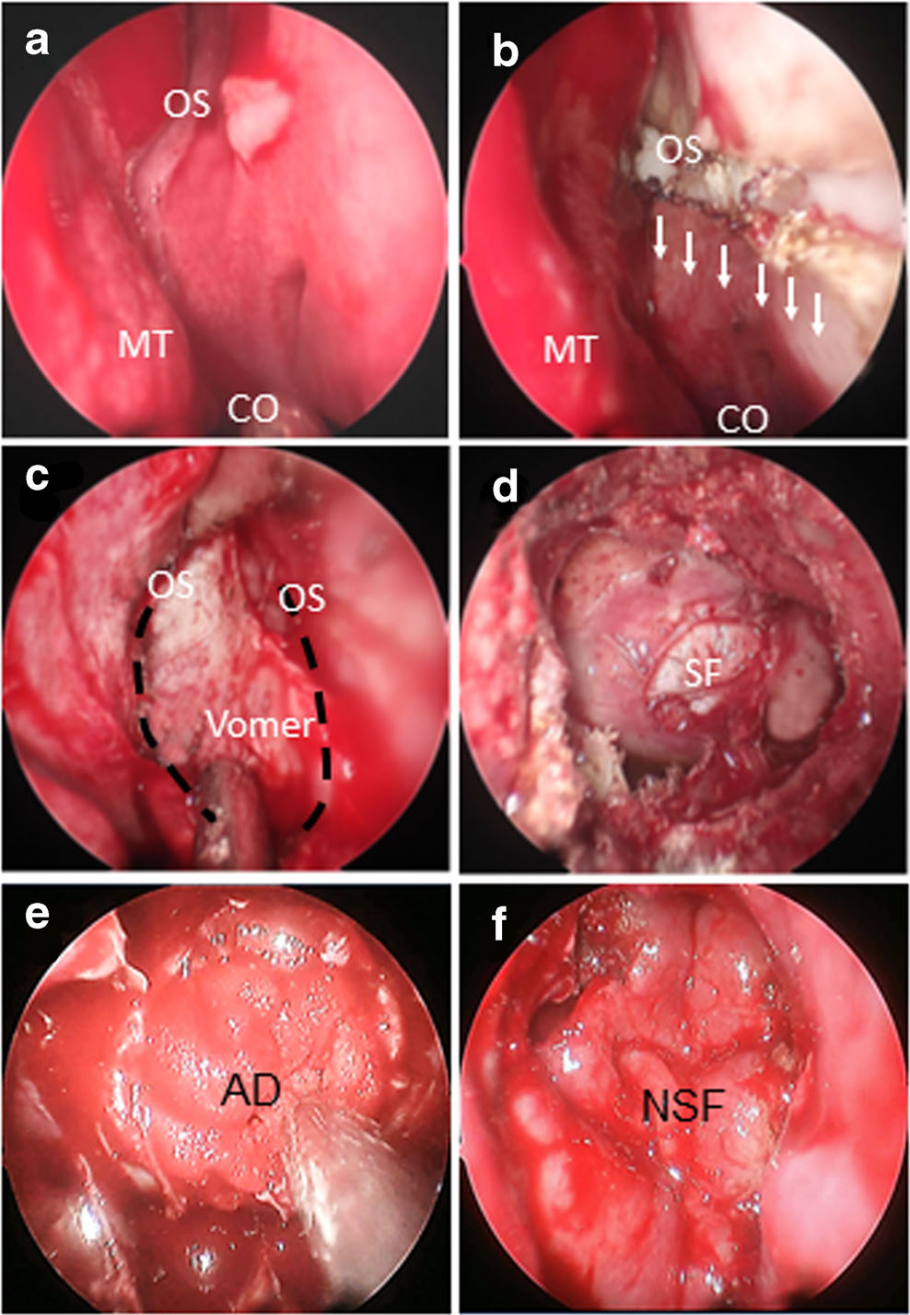
SOP of nasoseptal “rescue” flap technology: OS sphenoid sinus openings; CO choana; MT concha nasalis media; Vomer; SF sellar floor; AD artificial dura mate; NSF septal mucosal flap

**Fig. 2:**
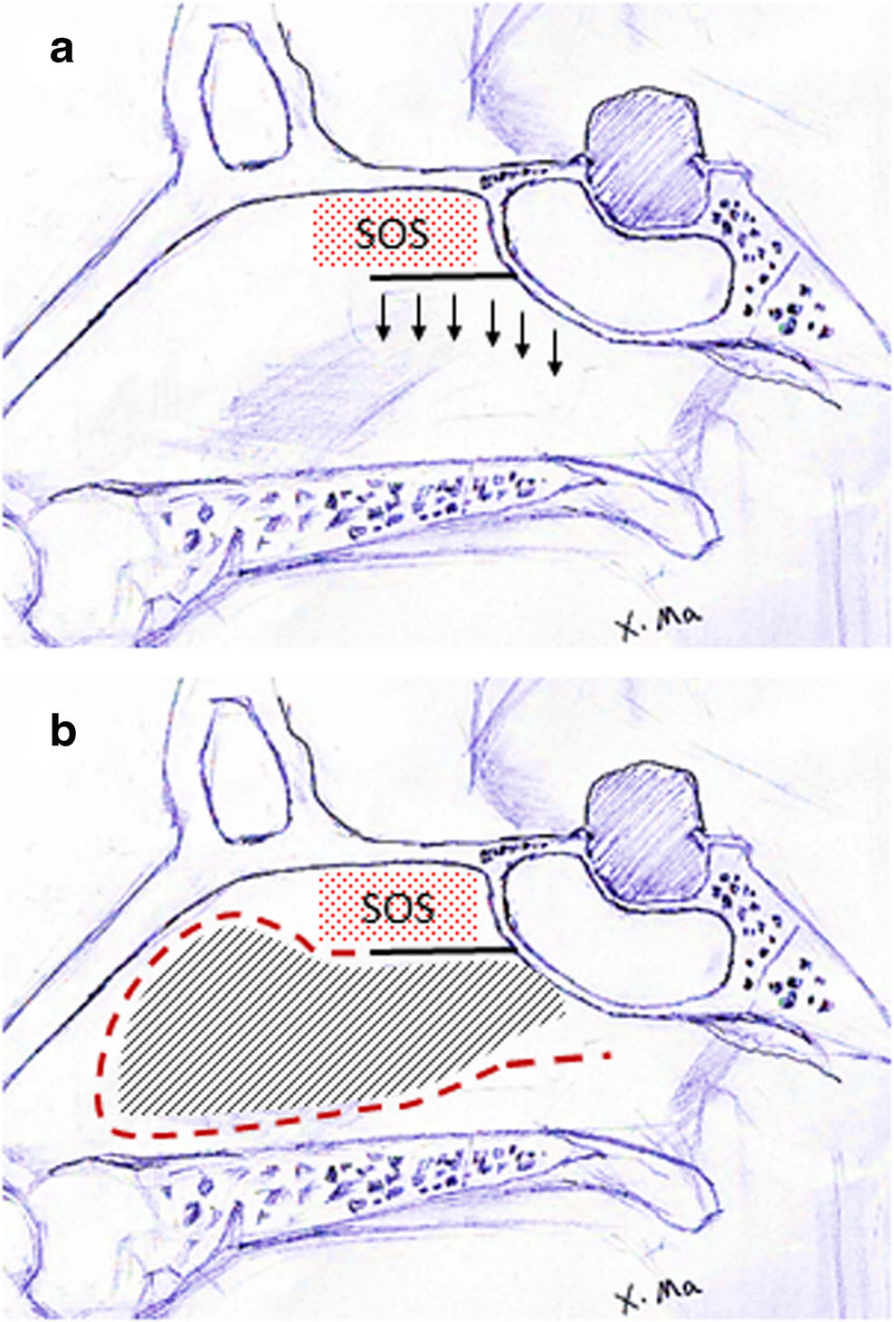
Schematic diagram of “rescue” flap. Red grid, olfactory region; black grid, black solid line, incision of “rescue” flap; red dotted line, incision of septal mucosal flap

1. Between January 2016 and March 2017, a total of 113 patients were treated with the EETSA of nasoseptal “rescue” flap technology at Qilu Hospital of Shandong University. Among them, male 51; female 62; age from 24 to 70, average is 50; tumor size < 0.5 cm 12, 0.5–5 cm 69, > 5 cm 32; non-functioning tumor 79, ACTH adenoma 13, GH adenoma 17, PRL adenoma 3, TSH 1 adenoma (Table 1).
2. The standard operation procedure of “rescue” flap technology:
  - Preparation of operation:
    i. Equipment of operating room: neurosurgical motorized operating bed, Xomed Power Instrument System (3-mm, 5-mm diamond drill), EEA system.
    ii. Surgical instruments: surgical EEA package, four direction angled ring curettes, curved suction, fine dissecting forceps.
    iii. Surgical consumables: disposable sterilized surgical dressings, surgical protective film, artificial endocranium.
    iv. 1% lidocaine with epinephrine in a 1/100,000 dilution to infiltrate nasal mucosa.
  - The position of patients during the operation:
    i. The patients were in supine position with the trunk elevated up to 20–30° to reduce the pressure of vein and bleeding. Patients’ head should be turned to surgeon’s direction and frontal-chin line should be at the horizon level in the meanwhile, adhesive bandage is necessary for fixation of the head if there is no head brace.
    ii. Mark the right lateral thigh with a straight incision about 3 cm long in preparation of fascia lata and fat when there is high fluid CSF leaking.
    iii. Sterilize patients’ face and thigh in common way and whisk sterile drapes on target area.
  - SOP of nasoseptal “rescue” flap technology:
    i. Povidone iodine solution irrigating both of the nasal cavity.
    ii. Infiltrated anterior nasal septum with epinephrine dilution under the conduction of 0° endoscope.
    iii. Select the right or the roomier side to conduct the endoscope, dilution. The middle turbinates are outfractured to facilitate visualization of the position of transsphenoidal opening, lateral abdominal wall, and posterior nasal cavity of lateral abdominal wall sinus septum usually with a detacher and epinephrine infiltrated brain cotton under it (Fig. 1a).
    iv. Incisions are performed following the sagittal plane of the septum from transsphenoidal opening to the lateral margin of the middle turbinate (monopolar electrotome, electrocoagulation mode, 20 w) (Fig. 1b; Fig. 2b).
    v. Elevation of the mucosa of the middle turbinate and sinus septum starts anteriorly with a detacher or similar instrument and also fracture the middle turbinate toward another side, revealing both side of the bony structure of lateral wall of sphenoid sinus and the opening of bilateral sphenoid sinus (Fig. 1c).
    vi. Remove the bony structure of lateral wall of sphenoid sinus with diamond drill, especially the sphenoidal rostrum and posterior nasal septum bone. Expand the exposure to facilitate intraoperative operation and remove the inner mucosa of sphenoid sinus and expose the sellar floor (Fig. 1d).
    vii. Expose the base of the saddle and remove the tumor.
    viii. If the intraoperative saddle septum is intact during the operation, no cerebrospinal fluid outflow. To fill the operated area with surgical fibers, we then use artificial dura mater to reconstruct the sellar floor and gelatin sponge to fill the sphenoid sinus as well as restore the “rescue” flap to the right position (Fig. 1e).
    ix. If there was low-flow CSF fistula during the operation, we recommend that the incision of “rescue flap” should be prolonged to the junction of mucosa and skin besides cover the operated area with artificial dura mater (Fig. 2b) and osteocomma obtained from the middle turbinate (Fig. 2b).
    x. If high-flow cerebrospinal fluid leakage occurs during the operation (large diaphragma sellae rupture or the cistern of the brain is wide open even the base of the third ventricle), we should use thigh fat and fascia lata to fill the dura, and cover nasal septum mucosa flap to the epidural and peripheral bone, then fill sphenoid sinus with gelatin sponge as well as the nasal cavity with oil gauze.

**Table 1:** Patients’ demographics

Clinical Data(2016.01–2017.03)	Patients’ using “rescue” flap (113 cases)
Age	24–70 (50 ± 11)
Gender	
Male	51
Female	62
Tumor size	
Microadenoma	12
Macroadenoma	69
Giant adenoma	32
Types of tumor	
Non-functioning adenoma	79
ACTH adenoma	13
GH adenoma	17
PRL adenoma	3
TSH adenoma	1
Average time in nasal cavity and sphenoid sinus	15–30 (24 min)
CSF fistula	27
Low-flow CSF fistula	24
High-flow CSF fistula	3
Transmit to nasal septum mucosa flap	23
Complication related to nasal cavity	
Anosphrasia	17
Epistaxis	1
CSF rhinorrhea	1

## Results

The duration of nasal and sphenoid sinus stage was 15–30 min, averaging 24 min. There were 27 cases of intro-operative cerebrospinal fluid leakage, including 24 cases of low-flow cerebrospinal fluid leak and 3 cases of high-flow cerebrospinal fluid leak. Twenty-three cases were converted from nasoseptal “rescue” flap to nasal septum flap as there was CSF fistula occurred during the operation in these patients. There were 17 cases of postoperative olfactory decline or disappearance, 1 case of epistaxis and 1 case of cerebrospinal rhinorrhea.

## Discussion

Cerebrospinal fluid rhinorrhea is one of the main complications of surgical treatment of pituitary adenoma via transsphenoidal approach under neuroendoscope. Surgeons used to apply autologous fat to fill in the leak (bath plug method), and then covered with fascia lata. However, this flap could not be converted without compromising the blood supply, which may lead to postoperative local transplantation of fascia necrosis or absorption, resulting in failure of repair and cerebrospinal fluid nasal fistula, and this method requires a separate incision in the thigh or abdomen, aggravating the trauma and psychological burden of the patient. The nasoseptal “rescue” flap technique reduces the incidence of the complications and represents an intuitive extension of neurosurgeons’ experience with the endoscopic resection of giant pituitary adenoma, craniopharyngioma, and saddle nodules meningioma using traditional external approaches as well as our experience with the reconstruction of the skull base after oncologic resections [1, 4–6].

For the giant pituitary adenoma expanding toward to suprasellar regions and cavernous sinus, surgeons could consider the application of extended transsphenoidal approach, which means remove the concha nasalis media of approaching side at the beginning of operation and prepare rescue flap well at choana for the reconstruction of base skull as well as a wider space for EEA operation [5] [3]. At present, the common methods of endoscopic transnasal transsphenoidal pituitary adenoma resection for the treatment of lateral wall and nasal septum mucosa of sphenoid sinus include electrocautery pneumatolysis sphenoidal mucosa which is at the opening of sphenoidal sinus (Fig. 4a, b), small mucosal flap around the opening of sphenoidal sinus (Fig. 4c, d), submucosal approach of nasal septum (Fig. 4e, f), and nasoseptal “rescue” flap. The first two approach applications are limited for usually affecting the blood supply from palatal artery and creating septum mucosal flap pedicle to repair CSF fistula. The incision between the nasal septum mucosa and epidermal junction is conducive to protect the nasal mucous membrane, but relatively narrow for operative space, which during the operation requires extensive stripping nasal septum mucosa and removing the septum bone. For the hypophyseal adenoma without CSF fistula, this approach would induce unnecessary trauma, prolong operation time, and increase the incidence of nasal discomfort. So nasal rescue flap has provided us with a fast and convenient way to enter the sphenoid sinus, and created conditions for the production of nasal septal pedicled mucosal flap for possible cerebrospinal fluid leakage.

**Fig. 3:**
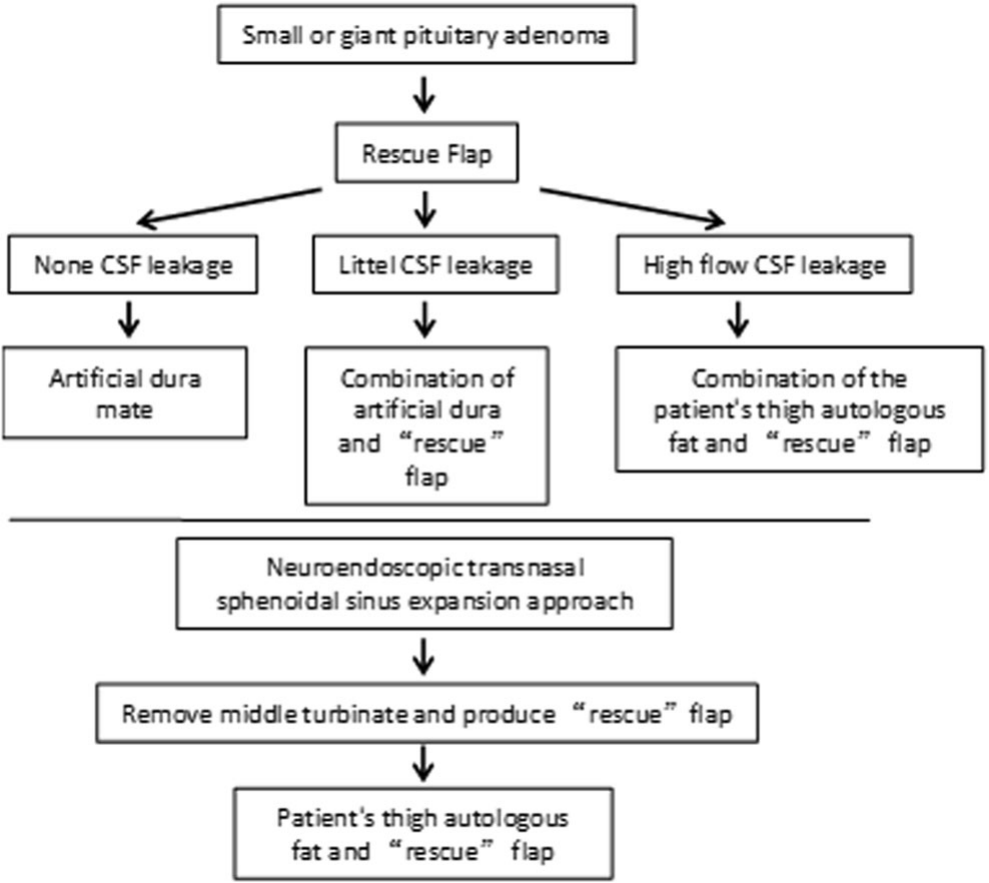
Strategy of sellar floor reconstruction in pituitary adenoma resection by transnasal transsphenoidal approach under neuroendoscope in Qilu Hospital

**Fig. 4:**
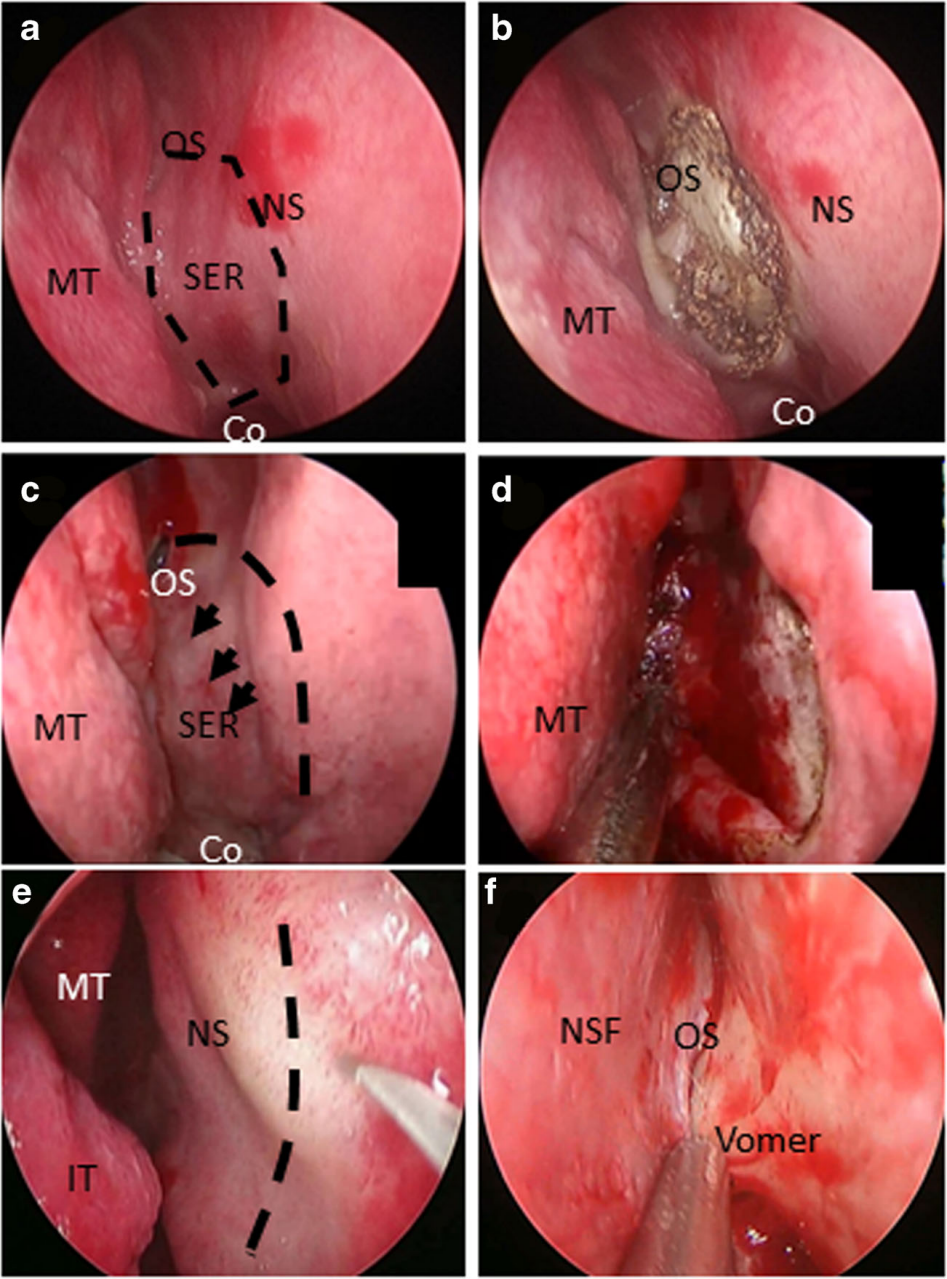
The common strategies of treating the lateral wall of the sphenoid sinus and the septum mucosa endoscopic transnasal transsphenoidal approach for pituitary adenoma resection. Electrocautery cavitation at the opening of the sphenoid sinus (**a**, **b**). Coverage with the small mucosal flap originated from vomer (**c**, **d**). Submucosal approach of the septum (**e**, **f**)

The application of mucosal rescue flap technology into sphenoid sinus is simple and rapid; the average time in our cases is 24 min (15–30 min). At present, there was few relevant literature report on olfactory function evaluation after endoscopic transsphenoidal pituitary adenoma resection. Rotenber reported that 36% of patients reported varying degrees of anosmia, and in our cases, 17 patients experienced anosmia [7, 8]. The incision should be horizontal to the level of the sphenoid sinus opening to avoid the olfactory area near the sphenoid recess and the upper turbinate (Fig. 2), thus decreasing the anosmia after operation [7, 8]. Here, we want to share some cases with this technique: one patient with ACTH microadenoma developed CSF fistula during the operation, as the leakage flow was small, we used single artificial dura to repair the leakage while no septal mucosal flap was made to strengthen the sellar floor. Two weeks after surgery, the patient’s nasal cavity was observed to have cleared the fluid outflow. Re-examination with endoscope showed that there was cerebrospinal fluid exudation at the bottom of the saddle. This case warned us that there was no fluke during the operation of pituitary adenoma under neuroendoscope; every single leakage should also be repaired with the “rescue” flap. So, our group now follows these principles of sellar floor reconstruction during the operation of pituitary microadenoma, large adenoma (Figs. 3 and 4): (1) if the diaphragma sellae was intact, and no cerebrospinal fluid exudation was observed after increasing the pressure at the end of exhalation, just use single artificial dura to reconstruct the sellar floor; (2) for the low fluid CSF fistula, the combination of artificial dura and “rescue” flap should be used; (3) for the high fluid CSF fistula, we need to take the patient’s thigh autologous fat and broad fascia to fill the saddle, and then use the septum mucosa flap to reconstruct the sellar floor. The operation of pituitary adenoma should be based on the concept of safety, efficiency, and minimally invasive, among which safety should be at the first place in all. While surgeons striving for minimally invasive, the occurrence of postoperative nasal leakage should be avoided as much as possible.

To sum up, the application of “rescue” flap could efficiently reconstruct the sellar floor when there is CSF fistula during the operation with no obvious increasing of the operative time and the incidence of postoperative olfactory loss. So, it can be used as a routine technique for endoscopic transnasal sphenoidal approach to treat the septal mucosa at the opening of sphenoid sinus.
